# Knowledge about COVID-19 and pandemic impact on Madrid dental students (Spain)

**DOI:** 10.4317/jced.58005

**Published:** 2021-08-01

**Authors:** Isabel Leco-Berrocal, José F. Martín-Morales, Isabel F. Tresguerres, Rosario Garcillán-Izquierdo

**Affiliations:** 1 DDS, PhD. Associate Professor. Department of Dental Clinical Specialties, Faculty of Dentistry, Complutense University of Madrid, Spain; 2 MD, DDS, PhD. Assistant Professor. Department of Dental Clinical Specialties, Faculty of Dentistry, Complutense University of Madrid, Spain; 3 MD, DDS, PhD. Associate Professor. Department of Dental Clinical Specialties, Faculty of Dentistry, Complutense University of Madrid, Spain

## Abstract

**Background:**

COVID-19 has had a major impact on dental activity, with implications on dental education. The aim of this study was to assess the knowledge about it and the pandemic impact on Spanish dental students.

**Material and Methods:**

An observational and cross-sectional study with a 17-items questionnaire was conducted. It was divided into three sections, sociodemographic data, self-perception and knowledge about the COVID-19 protective measures and repercussions on dental students. This survey was carried out in May 2020, and the response rate was 46.16%. The statistical analysis was performed by the Pearson chi-square test and Fisher’s exact test.

**Results:**

A total of 235 students responded to the questionnaire, with an average age of 22.3 years. Attendance at COVID-19 training courses, knowledge about the Personal Protective Equipment (PPE) management and the incubation period had the worst results, compared to the knowledge about hand washing, wearing gloves and masks and symptomatology who got higher percentages. 31.1% of participants reported presenting symptomatology related to SARS-CoV-2, although only 8.2% were diagnosed and 46.6% suffered quarantine. 62.5% were afraid to catch the disease.

**Conclusions:**

The results of this study show that there are deficiencies in the knowledge of important aspects of COVID-19 in dental students, which implies a commitment of the university in its training, as well as the realization of diagnostic controls for the disease.

** Key words:**Dental students, SARS-CoV-2, COVID-19, survey, knowledge.

## Introduction

The infectious disease caused by the SARS-CoV-2 coronavirus (known as COVID-19) started in Wuhan – China- in December 2019, and it has become a major public health problem not only for China, but also for a large number of countries around the world (1). On January 30, 2020, the World Health Organization (WHO) declared it a public health emergency of international interest (2).

In Spain, the first case of COVID-19 was reported on January 31, 2020 and until October 5 there were 813.412 confirmed cases and 32.225 deaths due to this infection (Mº Sanidad, actualización nº 221. Enfermedad por el coronavirus (COVID-19). [2020]. https://www.mscbs.gob.es/en/profesionales/saludPublica/ccayes/alertasActual/nCov/documentos/Actualizacion_221_COVID-19.pdf ). This situation caused the declaration of the state of alarm in our country from March 14 (Bol. Of. Del Estado. 14th of March 2020:25390-25400) to June 21, that followed to take preventive measures, such as home confinement and social isolation with significant economic, social and health repercussions that have yet to be determined. And despite global efforts to contain the viral spread, the outbreak has not yet stopped.

SARS-CoV-2 is transmitted between people through respiratory drops and aerosols (coughs and sneezes) and by direct contact with an infected person or a contaminated surface and transferring it to the mouth, nose or eyes (3). This disease has an incubation period that extends from 2 to 14 days with varying severity from asymptomatic clinic to life-threatening symptoms among different individuals (4). Infected people may be asymptomatic, and therefore these people make it difficult to diagnose and prevent transmission of the disease (5).

For this reason, it is considered that, among healthcare personnel, dentists seem to have a high risk of exposure to SARS-CoV-2. Odontology practice has been severely compromised by the pandemic (6) however, dental studies do not top the list of priorities of government agencies when planning crisis management of health care systems for the effective reorganization of dental care services, educational and research (7). Nevertheless, dentists have a relevant role, as they and their staff are familiar with infectious diseases (HIV or hepatitis) and used to work in controlled environments (8).

In turn, the COVID-19 pandemic has had a dramatic impact on dental education, the closure of dental clinics for students has meant a shift towards a fully virtual dental education with theoretical teaching not be done in person, conducting to long term repercussions that may affect clinical practice, dental education and research (9). In this sense, Bennardo at al. (10) reported that blended learning will be probably the cornerstone of future dental education, with clinical rotations, which will be organized according to the clinical needs and safety of the dental staff.

The arrival of the next academic year and the opening of faculties of Dentistry, ensuring academic and research continuity, involves redesigning the infrastructures, while occupational risks are managed, guaranteeing safety for patients, students and staff. The recovery period can be long, because to date there is no vaccine for SARS-CoV-2, which implies that the application of preventive measures to control the infection is the most important intervention, at the same time that teachers must guarantee the maintenance of a quality education and preserve the attention of oral health of the patients (11).

For this reason, this work was carried out with the aim of evaluating the knowledge and self-perception of dental students about COVID-19, as well as to study the situation experienced by students during the pandemic period, in order to facilitate the incorporation into face-to face teaching, and to know the needs of the students for the present course.

## Material and Methods

An observational and transversal study was carried out at the Faculty of Dentistry at Complutense University, in Madrid, Spain. An online survey was designed, trough Google Forms. This survey was sent to 509 undergraduate students who were registered in 2019-20 year in the aforementioned university. These students signed the consent form prior to their enrollment. The survey run from 22 May until 29 May 2020, with a participation rate of 46.16% of all students.

The study protocol was approved by the Ethics Committee at San Carlos Hospital, Madrid, and was performed following the Helsinki Declaration, emphasizing the anonymity of participants.

First part of the questionnaire consisted of an introduction and objectives of the study. In this introduction, the voluntary participation and the confidential treatment of data were remarked. Then, seventeen questions were performed divided into three sections:

1. Sociodemographic data: age, gender and course.

2. The knowledge and self-perception of COVID-19 control were evaluated by six items, with three possible answers (yes, no, not enough). Conference attendance, webinars and other kind of courses about COVID-19 were registered, and also the knowledge about hand hygiene, gloves, mask and personal protective equipment (PPE) using. Clinical symptoms, and SARS-CoV-2 incubation period were also reported.

3. The last part was related to the personal situation during the pandemic period. Seven questions of multiple choice were inserted, including presence of symptoms, type, duration, need of quarantine, and if it was so, its cause, exact diagnosis confirmed by PCR, direct contact with a confirmed patient or a suspected patient, antibodies testing, etc. Finally, the general perception of fear of getting the disease, was registered in a 5-point Likert-type scale: 1= never, 2=seldom, 3= sometimes, 4= very often, 5= always.

To analyze the data, a distribution following the age, gender and course was performed. Three groups were considered for the variable “age”: 18-20 years, 21-25 years, and more or equal than 26 years. Respect to the symptoms, three groups were taken into account: 1-3 symptoms, 4-5 symptoms or more or equal than 6.

Data were registered in an excel Table, showing frequency and percentage. The statistical analysis was performed by the Pearson chi-square test and Fisher’s exact test. Statistical significance was considered when *p*<0.05. All analyses were performed using SPSS 23.0.

## Results

235 under-graduate students of Dentistry were participants in this study, representing 46.16% of all students. Mean of age was 22.3 ± 4.3 years. The female participation rate was 78.7%. Thus, the ratio female to male students was 3.7 to 1. The graduate courses with more participation were fourth year (26.4%), first year (23.4%) and fifth year (20.4%), in this order (Table 1).

**Table 1:**
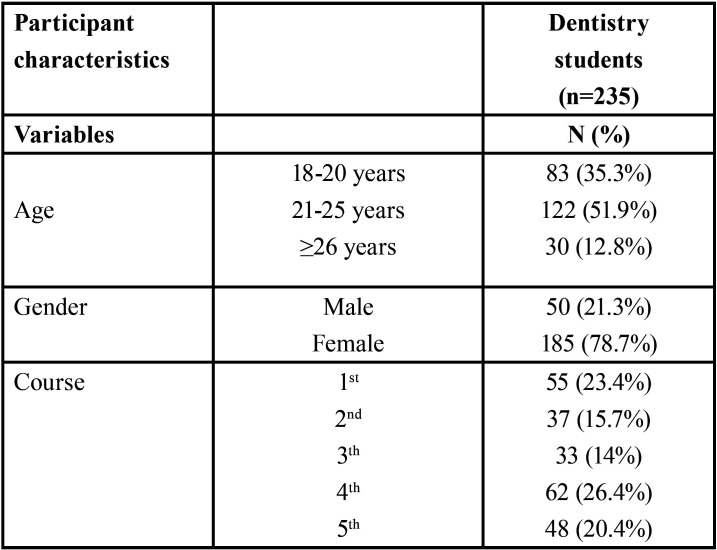
Description of sociodemographic data.

The knowledge and formation of students in COVID-19 presented differences among the age of the groups, gender and course (Table 2).

**Table 2:**
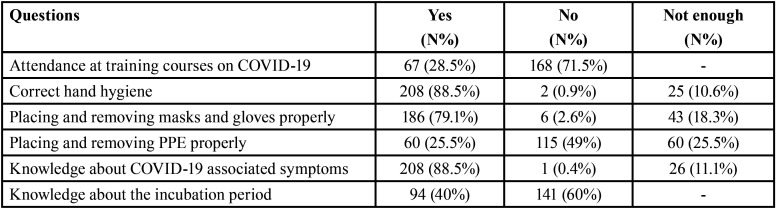
Results of knowledge and training questions.

Students who were more worried about their formation were the group ≥ 26 years old and fifth course (63.3%), in front of their classmates of 21-25 years old (34.4%), 18-20 years old (6%), and respect to the lower grades, being the differences statistically significant (*p*<0.001). Male students were who most formation received (46%) respect to the female (23.8%), and the differences were statistically significant (*p*<0.05).

The lack of knowledge about PPE management was superior in the 18-20 years old group (59%) and the female group (52.4%), in front of the ≥ 26 years old group (13.3%) and the male group (36%), being the differences statistically significant (*p*<0.05).

No differences were found neither the knowledge about hands hygiene, gloves and mas 

management, nor the questions about symptoms and incubation period of SARS- CoV-2.

**Figure 1:**
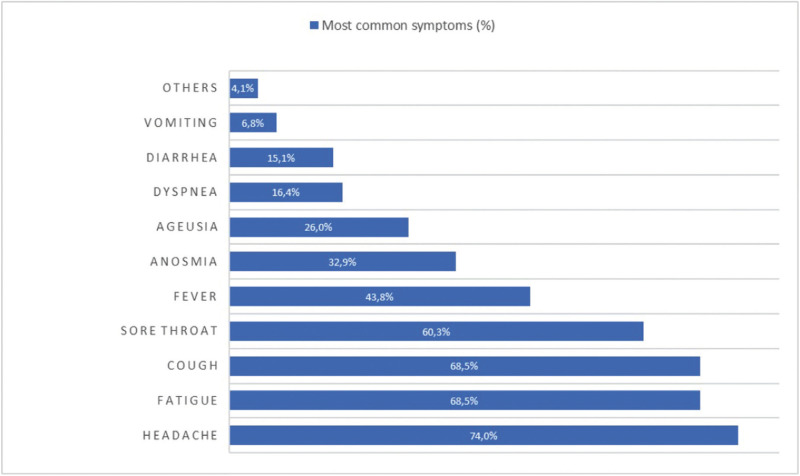
Symptoms referred by participants.

The third block data showed that 31.1% (n=73) of the participants presented symptoms

related with COVID-19 (Table 3). The most frequent symptoms were headache, fatigue, coughing, throat pain, fever, anosmia, ageusia, dyspnea, diarrhea, and vomiting, as reflected in Figure 1.

**Table 3:**
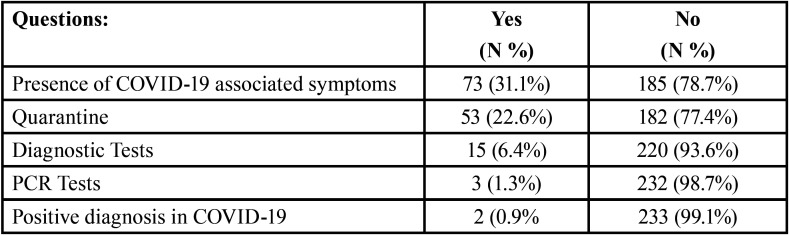
Results of the COVID-19 impact on dental students.

The mean of symptoms of each participant was 4.2 ± 1.4. 45% of all participants presented 1-3 symptoms, while 31.5% showed 4-5 symptoms, and 23.3% had 6 or more symptoms. The more frequent association of symptoms was fatigue, headache and coughing (35.6%), and the second one was fatigue, fever and coughing (26%).

No relation between age and presence of symptomatology was detected, but male students presented significantly more throat pain and fever than female (*p*<0.05).

Among the symptomatic students, six (8.2%) were subjected to diagnostic tests, including PCR (n=2). No statistically significant differences were found respect to the no symptomatic group. 53 participants followed quarantine (22.6%); 90.6% of them responded the reason: 57.5% were suspected of COVID-19, 40.7% were related with a confirmed case, and only 1.8% with confirmed diagnosis. The 46.6% of the symptomatic participants followed quarantine in front of 11.7% of no symptomatic, existing a statistically significant relation between the presence of symptoms and the number of them, and the compliance with the quarantine requirements (*p*<0.001).

Regarding the students who had fear of getting the COVID-19, the data reflected in Table 4, show that 62.5% were sometimes or always afraid of getting the infection, in front of 37.5% who were never afraid or almost never. The fear was more pronounced in the female and in the quarantine groups (*p*<0.05). No statistically relation were found in the symptomatic students regarding to the fear, although more symptomatic were always afraid (16.4%), respect to the no symptomatic (9.3%).

**Table 4:**
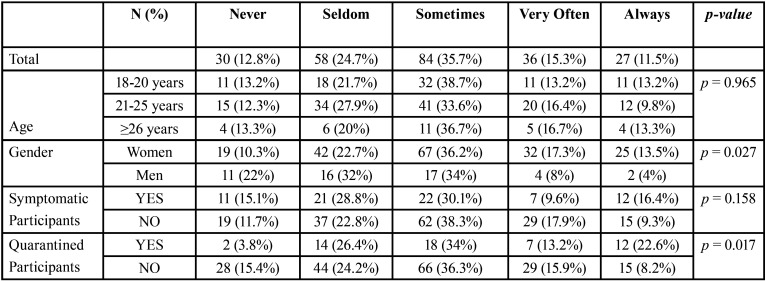
Results of participants’ fear to get the disease.

## Discussion

Dental procedures can produce dissemination of contaminants from saliva, blood and other body fluids, as well as the use of sharp instruments can conduce to a risk of cut, which implies that dental professionals are more likely to get infected by SARS-CoV-2 or to spread it to their patients (8,10). WHO recommends that, as far as transmission rate is reduced, only absolutely essential dental treatments will be applied; thus, the maintenance of the bucodental function or to avoid acute pain, ensuring quality of life, could prevent patients from going to a hospital, freeing up the emergency service (WHO. Interim guidance. Considerations for the provision of essential oral health services in the context of COVID-19. [2020]. https://www.who.int/publications/i/item/who-2019-nCoV-oral-health-2020.1). In this sense, dental clinicians and Faculties of Dentistry could act in the first line of detection of systemic diseases, including COVID-19; here lies the true importance of qualification not only for professionals, but also for students (8).

Thus, this study can be considered of interest because the aim of this work is to evaluate the students´ knowledge about COVID-19 and the pandemic impact on students of Dentistry, in order to make improvement plans to address the face-to-face teaching.

The results have shown that only 28,5% of surveyed students confirmed that they had trained on COVID-19, on the date of the survey. This percentage is lower than Bhagavathula *et al*. (11) reported in March among healthcare professionals all over the world. In this paper, 44.1% of respondents had recognized their training on COVID-19 by conferences and debates. It should be emphasized that students in their last course or those who were older were the most trained. This can be justified because students ≥ 26 years old are already working in jobs related to health sciences, and 5th course students are on the brink of starting their professional working life.

The hand hygiene is considered as one of the key interventions for minimizing the risk of microorganism transmission (12), playing a crucial role for prevention the SARS-CoV-2 spread among healthcare professionals. However, some reports have shown that handwashing is often neglected by healthcare professionals, not only in underdeveloped countries, but also in developed ones, finding poor compliance with this practice (13).

In the current study, 88.5% of students reported that they had the knowledge about the handwashing procedure, in agreement with Modi´s *et al*. data (14), although they did consider the question “Five moments for hand hygiene”, described by WHO (15), and conducted the survey not only among students and teachers, but also Dentistry and other Health Sciences professionals and administrative staff. In a previous study, Modi *et al*. (16), concluded that Medicine students must receive an adequate washing hands training from the first year of studies, through annual workshops, becoming in a requirement for their clinical skills evaluation.

All dental staff must be trained in effective use of the PPE, referring to protective gloves and impermeable gown, glasses, face shields and masks, which must protect mouth and nose by a device designed to achieve a very closed facial fit, such as surgical N95 respirators (WHO. Interim guidance. Rational use of personal protective equipment for coronavirus disease (COVID-19) and considerations during severe shortages. [2020] https://www.who.int/publications/i/item/rational-use-of-personal-protective-equipment-for-coronavirus-disease-(covid-19)-and-considerations-during-severe-shortages). In this sense, 79.1% of the participants in this last study corroborate that they knew how to wear the gloves and the mask, however, only 25.5% recognized to be competent in the proper use of the PPE. These are unfavorable data when compared with the study performed in India, in which 67% of the surveyed dentists said that they had a reasonable knowledge about PPE (17).

Regarding knowledge of the symptoms associated with COVID-19, 88.5% of participants claim to know them, coinciding with the above-mentioned survey (17), however only 40% correctly answer the question about the incubation period. In this sense, it should be noted that asymptomatic patients and during the incubation period are a source of transmission of the disease, and considering that many of these patients may be likely to need dental treatment, it’s essential to determine a safe period of time to treat suspicious patients, therefore the importance of their knowledge by dental professionals and students (18).

At the same time, it is necessary to take into account the different situations experienced by the participants with respect to COVID-19, the need for social distancing and isolation along with uncertainty about the future of the pandemic, that can significantly affect students’ mental health, their learning and academic performance (19).

The results of this work show an incidence of related symptomatology with SARS-CoV-2 in 31.1% of the sample, of which 46.6% made quarantine and only 8.2% diagnostic tests and within them 2.7% PCR, which were positive. In this regard, it is necessary to insist on the importance of diagnostic tests for rapid and accurate detection, and to monitor community and hospital outbreaks (20). WHO indicates molecular testing (e.g. RT-PCR) of respiratory tract samples is the recommended method for identification and laboratory confirmation of cases of COVID-19, prioritizing its use compared to other strategies, such as antibody-based testing that provide information from the recovery phase and that can give false positives, and rapid detection tests for antigens or antibodies that are not considered suiTable for the diagnosis of an acute infection (Mº Sanidad, instrucciones sobre la realización de pruebas diagnósticas para la detección de COVID-19 en el ámbito de las empresas. [2020] htpps://www.mscbs.gob.es/profesionales/saludPublica/ccayes/alertasActual/nCov-China/documentos/instruccionesPruebasDiagnosticasEmpresas.pdf).

Quarantine to control infectious diseases is a practice widely documented for centuries. Today, many countries have the legal authority to impose quarantine which, in accordance with Article 3 of the International Health Regulations (2005), must be applied with full respect for the dignity, human rights and fundamental freedoms of individuals WHO, International Health Regulations. [2005] htpps://www. apps.who.int/iris/bitstream/handle/10665/246107/9789241580496-eng.pdf?sequence=1). In the COVID-19 environment it is the restriction of activities or the separation of people who are not sick but could have been in contact with an infected person (SSHAP, Key considerations: quarantine in the context of COVID-19. [2020]. https://www.socialscienceinaction.org/resources/february-2020-social-science-humanitarian-action-platform/). The goal is to keep them under observation in case of symptoms and ensure early detection of cases of COVID-19. But social distancing can in turn lead to anxiety disorders that worsen in the absence of interpersonal communication (21). That along with dubious or even false information about transmission, incubation period, geographical scope, number of infected, actual mortality rate, inadequacy of control measures and therapeutic mechanisms, increase insecurity and fear in the population (22).

Public health emergencies can have many psychological effects in college students, that can be expressed as anxiety, fear and worry, among others, and require attention, help and support from the society, families and university (23).

Martínez-Lorca *et al*. (24) in his survey done with Spanish university students, achieved a medium or moderate level (FCV-195 scale) of fear of COVID-19. These results are not comparable to those obtained in this study, where a scale was not used, only one question was asked to which 67.5% of participants claimed to be always or almost always afraid to contract the disease. However, the results obtained by Alawia *et al*. (25) in dental students showed that 82% prefer to avoid working during the pandemic, because they themselves are not consider prepared and for fear of transmitting the virus to their family or friends.

## Conclusions

This study concludes that it is necessary to improve the training of dental students through courses, seminars or workshops, and even be part of the agenda of some subjects such as prevention, to make them have greater knowledge about the characteristics of the virus, epidemiology, as well as preventive measures in the dental cabinet.

In turn, it should be noted that a third of students have had symptoms related to COVID-19, although on the date the survey was conducted, a low percentage of colleges had undergone diagnostic or serological tests. Therefore, it is recommended that all students who perform clinical treatments on patients be subjected to immunity checks.
